# Genome-Wide Identification and Structural Characterization of Growth-Regulating Factors (GRFs) in *Actinida eriantha* and *Actinidia chinensis*

**DOI:** 10.3390/plants11131633

**Published:** 2022-06-21

**Authors:** Muhammad Abid, Zupeng Wang, Chen Feng, Juan Luo, Yi Zhang, Jing Tu, Xinxia Cai, Puxin Gao

**Affiliations:** 1 Lushan Botanical Garden, Chinese Academy of Sciences, Jiujiang 332900, China; fengc@lsbg.cn (C.F.); luojuan0118@163.com (J.L.); yitea7@163.com (Y.Z.); tujing0819@163.com (J.T.); cai_xinxia20220304@163.com (X.C.); 2 Key Laboratory of Plant Germplasm Enhancement and Specialty Agriculture, Wuhan Botanical Garden, Chinese Academy of Sciences, Wuhan 430074, China; wangzupeng@wbgcas.cn

**Keywords:** kiwifruit, *GRF* gene family, *A. chinensis*, *A. eriantha*, gene structure, synteny analysis

## Abstract

Growth-regulating factors (*GRFs*) encode plant-specific transcription factors that play a vital role in regulation of plant growth, development, and stress response. Although *GRFs* have been identified in various plants, there is no reported work available in *Actinidia* (commonly known as kiwifruit) so far. In the present study, we identified 22 *GRF* genes on *A. chinensis* (hereafter *A. chinensis* is referred to as Ac, and *GRF* genes in *A. chinensis* are referred to as *AcGRF*) distributed on 17 chromosomes and one contig, and 26 *GRF* genes in *A. eriantha* (hereafter *A. eriantha* is referred to as Ae, and *GRF* genes in *A. eriantha* are referred to as *AeGRF*) distributed on 21 chromosomes. Phylogenetic analysis showed that kiwifruit *GRF* proteins were clustered into five distinct groups. Additionally, kiwifruit *GRFs* showed motif composition and gene structure similarities within the same group. Synteny analysis showed that whole-genome duplication played a key role in the expansion of the *GRF* family in kiwifruit. The higher expression levels of kiwifruit *GRFs* in young tissues and under stress conditions indicated their regulatory role in kiwifruit growth and development. We observed two genes in Ae (*AeGRF6.1*, *AeGRF6.2*) and two genes in Ac (*AcGRF6.1*, *AeGRF6.2*) significantly upregulated in different RNA-seq datasets. The presence of conserved protein structures and cis-regulatory elements caused functional divergence in duplicated gene pairs. The subcellular localization indicated the presence of kiwifruit *GRFs* in the nucleus of the plant cell. Protein-protein interaction analysis predicted *AtGIF* protein orthologs for *AcGRFs* and *AeGRFs*. Taken together, we systematically analyzed the characterization of kiwifruit *GRF* family members for their potential role in kiwifruit development and *Pseudomonas syringae* pv. *actinidiae* (*Psa.*) invasion response. Further functional studies of kiwifruit *GRFs* in plant growth, development, and stress response will provide valuable insights for kiwifruit breeders.

## 1. Introduction

Transcription factors (TFs) are well known for their regulatory roles in plants. Growth regulating factors (*GRFs*) are plant-specific TFs that regulate growth, development, and abiotic stress tolerance [1]. The *GRF* gene was first reported in rice (*OsGRF1*) which encodes a protein for gibberellin to promote stem elongation [2]. Since then, there has been extensive work on the identification and evolutionary analysis of the *GRF* gene family in various plant species. So far, researchers have identified nine *GRF* members in *Arabidopsis thaliana* [3], 12 in *Oryza sativa* [4], 14 in *Zea mays* [5], 10 in *Brachypodium distachyon* [6], 30 in *Triticum aestivum* [7], 35 in *Brassica napus* [8], 22 in *Glycine max* [9], and 10 in *Jatropha curcus* [10].

Numerous studies on *GRF* members in plants demonstrated that there are two highly conserved domains (QLQ and WRC) present in the N-terminal. The QLQ (Gln, Leu, Gln, IPR014978) domain, present on the SW12/SNF2 protein, is specific for protein-protein interaction, while WRC (Trp, Arg, Cys, IPR014977) is a plant-specific domain that possesses a C3H motif for DNA binding [4,11]. In contrast to the conserved nature of the N-terminal, the C-terminal in *GRFs* is composed of variable amino acid residue and is responsible for transcriptional activation. Additionally, the C-terminal of *GRF* proteins also contains some less conserved motifs such as TQL (Thr, Gln, Leu) and FFD (Phe, Phe, Asp) [5,12].

Previous findings have demonstrated the involvement of *GRF* in plant growth and development. Members of the *GRF* family are highly expressed in young plant tissues indicating their regulatory role in plant tissue and organ formation [13]. *Arabidopsis* and rice *GRF* mutants exhibited dwarfism [14,15]. The rice plants overexpressing *GRF* exhibited increased growth parameters [16]. The *AtGRF7* interacted with *DREB2A* (dehydration responsive element-binding protein 2A) to regulate the osmotic stress response in *A. thaliana* [1]. Furthermore, the *GRFs* control the root growth, flower development, and size of seeds in plants [17]. The *GRFs* are regulated by miR396 to control the growth and development of plants [18]. Additionally, the *GRF-GIF* (*GRF*- interacting factors) transcriptional complex regulates the size of leaf and plant architecture [19].

The genus *Actinida* (kiwifruit), originating in the Yangtze river valley in China, is an economically important fruit plant comprised of 54 species and 75 taxa [20]. The economically important horticultural species within *Actinidia* include *A. chinensis* Planchon, *A. deliciosa* (*A. chinensis* var. *deliciosa* A. Chevalier), *A. arguta* (Siebold and Zuccarini) Planchon ex Miquel, and *A. eriantha* Bentham [21]. All *Actinida* species are perennial, deciduous, and dioecious with climbing or straggling growth habit. Most of the kiwifruit species are reticulate polyploids with a chromosome number of x = 29 [22]. The kiwifruit is gaining popularity among consumers owing to its high vitamin C, mineral contents, and vibrant colors of fruit flesh [23]. So far, there is no reported knowledge available on systematic investigation and functional analysis of the *GRF* gene family in kiwifruit. Therefore, the present study was designed to comprehensively analyze the structure and expression of *GRF* family members in previously reported whole genomes of *A. erinatha* (green-fleshed cultivar ‘White’, hereafter referred to as Ae) and *A. chinensis* (red-fleshed cultivar ‘Hongyang’, hereafter referred to as Ac) [24,25]. Current findings will provide valuable insights into the structure, function, and evolution of *GRF* family members in both kiwifruit species. 

## 2. Results

### 2.1. Identification of Kiwifruit GRFs

**Figure 1:**
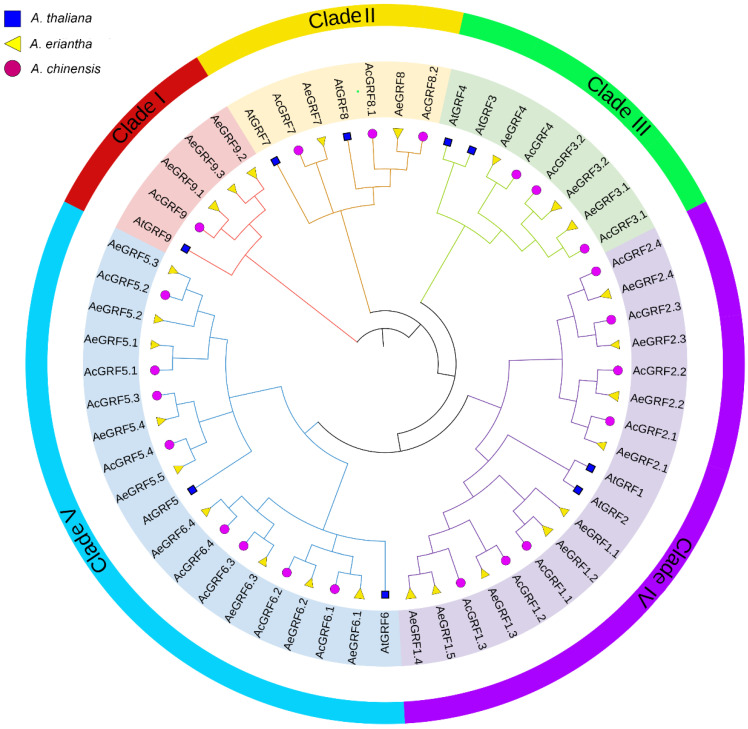
Phylogenetic analysis of *AcGRF* and *AeGRF* and *AtGRF* proteins. Each clade is represented by a different color. The circle shape denotes *AcGRF* proteins, the triangle shape represents *AeGRF* proteins and the square shape represents *AtGRF* proteins.

Based on the Hidden Markov Model (HMM) of WRC and QLQ domains, we identified a total of 26 proteins from Ae genome (*A. eriantha*, proteins named as *AeGRF*) and 22 proteins from Ac genome (*A. chinensis*, proteins named as *AcGRF*) (Figure 1 and Figure S1). The proteins in both kiwifruit genomes were named after their *A. thaliana* homologs. All the proteins contained both conserved domains except *AeGRF2.2*, *AeGRF1.2*, *AcGRF2.1*, *AcGRF9*, *AeGRF9.1*, *AeGRF9.2*, *AeGRF9.3*, *AeGRF5.1*, *AeGRF3.2*, and *AeGRF5.2*, which contained only one of the conserved domains. The coding sequences (CDS) length in *AeGRFs* and *AcGRFs* ranged between 522–1644 bp and 588–1773 bp, respectively. The length of putative *AeGRF* and *AcGRF* proteins were between 173–547 aa and 195–590 aa, respectively. The molecular weight (MW) of *AeGRF* and *AcGRF* proteins ranged between 19.42–59.45 kDa and 21.55–62.20 kDa, respectively. Moreover, the theoretical isoelectric point (pI) for *AeGRF* and *AcGRF* proteins varied between 5.8–9.69 and 6.31–9.70, respectively. The grand average of hydropathy (GRAVY) for *AeGRF* and *AcGRF* proteins ranged from −0.31 to −1.18 and −0.28 to −0.94, respectively (Table S1). The sub-cellular localization analysis predicted all kiwifruit *GRF* proteins localized in the nucleus of plant cells.

### 2.2. Sequence and Phylogenetic Analysis of Kiwifruit GRFs

All the kiwifruit *GRF* proteins contained one or both QLQ and WRC conserved domains or in their N-terminal regions (Figure S1). To gain insights into evolutionary relationships among kiwifruit *GRFs*, a phylogenetic tree was constructed by the neighbor-joining method for *AtGRF* (9), *AeGRF* (26), and *AcGRF* (22) proteins. All 55 proteins from different species were clustered into five clades (I–V) (Figure 1). Each clade contained proteins from all three species. Among five clades, clades I, II, and III were relatively small and contained five, seven, and eight proteins, respectively. By contrast, clade IV and V contained relatively large number of proteins (18 proteins in clades IV and 19 proteins in clade V). The phylogenetic tree suggested that kiwifruit *GRFs* showed a close relationship with *AtGRFs* partially because of the same dicotyledonous nature (Figure 1). 

### 2.3. Chromosomal Localization of Kiwifruit GRFs

**Figure 2:**
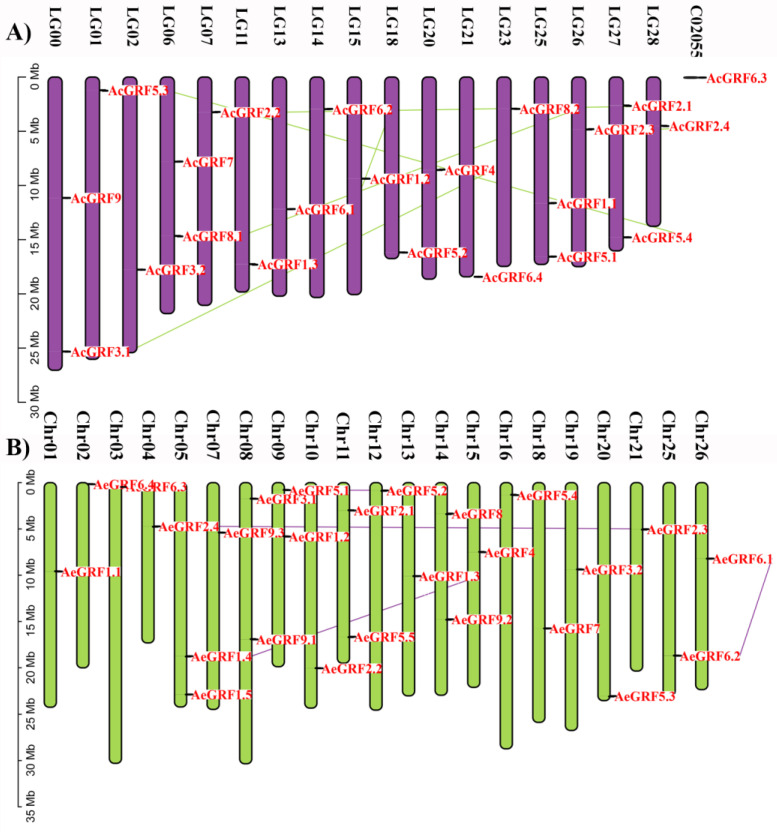
Chromosomal distribution of (**A**) *AcGRF* genes, and (**B**) *AeGRF* genes. The colored lines in the background show homologous gene pairs.

Kiwifruit *GRF* genes were unevenly distributed across the chromosomes (Figure 2). Results showed that 22 *AcGRFs* were distributed on 17 chromosomes and one contig. All the chromosomes contained only one gene except Chr 05, 08, 09, 11, and 14 which contained two genes on each chromosome (Figure 2A and Figure S2A). Similarly, the 26 *AeGRFs* were distributed on 21 chromosomes. All chromosomes contained only one gene except LG 00, 06, 25, and 27 which possessed two genes on each chromosome (Figure 2B and Figure S2B). 

### 2.4. Structural Analysis of Kiwifruit GRFs

**Figure 3:**
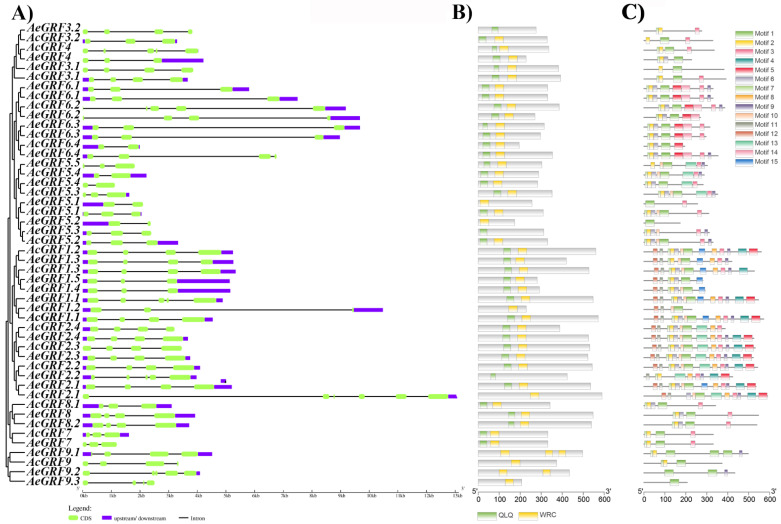
Structural analysis of kiwifruit *GRFs*. (**A**) Exon-intron analysis, (**B**) Identification of conserved domains, (**C**) Prediction of conserved motifs. The motif sequences are presented in Table S4.

We analyzed gene structure and motif characteristics to further explore the evolutionary relationship among kiwifruit *GRF* genes. The exon-intron analysis provides clues about the functional diversification of members of a gene family. The exon-intron number in kiwifruit *GRFs* ranged between 1–6. The *AcGR2.1* possessed a higher exon-intron number among all kiwifruit *GRFs* (Figure 3A and Figure S3). The MEME web server and Pfam database were employed to predict conserved domains and motifs in kiwifruit *GRFs*. All kiwifruit *GRFs* contained one or both QLQ and WRC conserved domain in their N-terminal region (Figure 3B). In total, 15 conserved motifs were predicted in kiwifruit *GRFs*. The maximum number of conserved motifs on single kiwifruit *GRF* ranged from 1 to 13. The *GRFs* belonging to the same clade have a similar motif composition. The clade III contained the maximum number of motifs, while clade I contained the minimum number. Additionally, some motifs appeared only in a specific clade. For example, purple and orange colored motifs are unique to clade III and V. Overall, structural analysis strongly supported the inferred phylogenetic relationships of kiwifruit *GRFs* (Figure 3C). The Logos for all conserved motifs in kiwifruit *GRFs* are presented in (Figure S4) and the sequences for motifs are presented in (Table S3).

### 2.5. Collinearity Analysis of Kiwifruit GRFs

**Figure 4:**
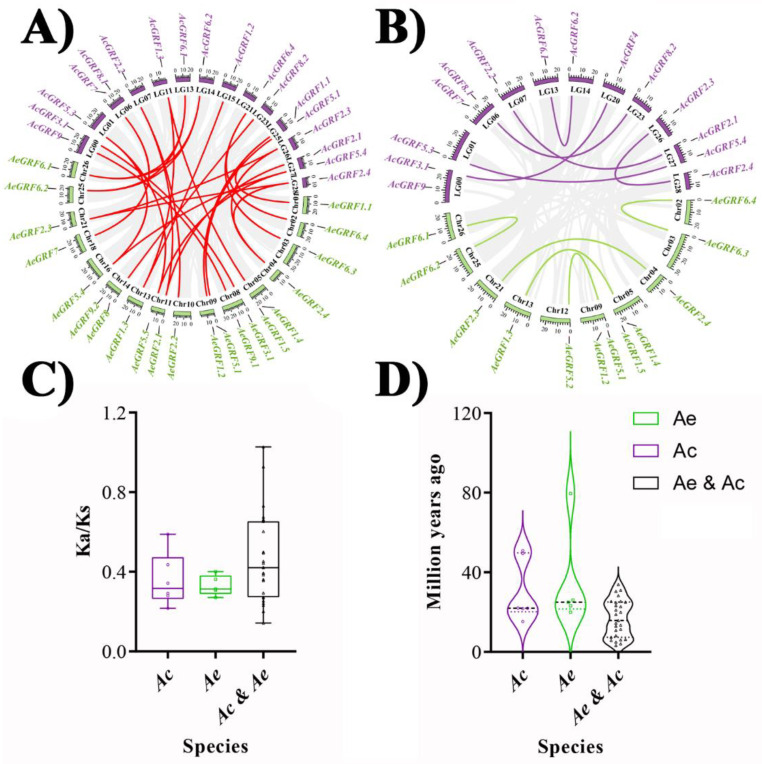
Collinearity analysis for kiwifruit *GRFs*. (**A**) Identification of ortholog gene pairs for Ae vs. Ac genomes. (**B**) Identification of paralog genes pairs for Ac vs. Ac genomes and Ae vs. Ae genomes. (**C**) Selection pressure calculation for *GRF* ortholog and paralog gene pairs between and within Ae and Ac genomes. (**D**) Calculation of divergence time (T). The grey lines in the background indicate all syntenic blocks between and within Ae and Ac genome, and colored lines indicate syntenic kiwifruit *GRFs* gene pairs.

To show the syntenic relationships, the whole genomes of Ac and Ae were aligned by Blastp to do an all-to-all blast and analyzed with MCScanX software for identification of syntenic blocks and duplicated gene pairs. The comparison between Ae vs. Ac genomes resulted in the identification of 27 ortholog gene pairs (Figure 4A). Similarly, the comparison between Ac vs. Ac resulted in six paralog gene pairs, and the comparison between Ae vs. Ae genomes resulted in five paralog gene pairs (Figure 4B). Gene duplication is an important phenomenon that forms a cornerstone for genetic novelty in plants and expansion or contraction of gene families. For an instance, over 90% of functional and developmental genes in *A. thaliana* have evolved by gene duplication [26]. The most common way to measure the ongoing adaptive evolution in genes is by calculating the ratio of non-synonymous (Ka) to synonymous (Ks) nucleotide substitution in duplicated gene pairs. We determined the selection pressure in duplicated gene pairs of kiwifruit *GRF* by calculating Ka, Ks, Ka/Ks, and T (divergence time). Generally, Ka/Ks >1 indicates positive selection, Ka/Ks = 1 represents neutral selection, and Ka/Ks < 1 denotes purifying selection [27]. Our results showed Ka/Ks < 1 for all paralog and ortholog gene pairs except *AeGRF5.5*/*AcGRF5.4*, suggesting that purifying selection was the main source of evolution for the kiwifruit *GRF* family (Figure 4C). The divergence time for *AcGRF* and *AeGRF* paralog gene pairs ranged between 15.25–50.87 and 19.97–79.55 MYA, respectively. Similarly, the divergence time for ortholog gene pairs between Ac and Ae *genomes* ranged from 3.29 to 33.83. Additionally, the divergence time for *GRF* ortholog gene pairs between Ac and Ae genomes was the smallest, while the divergence time of *GRF* paralog gene pairs in Ae was the greatest (Figure 4D and Table S2).

### 2.6. Expression Analysis of Kiwifruit GRFs

**Figure 5:**
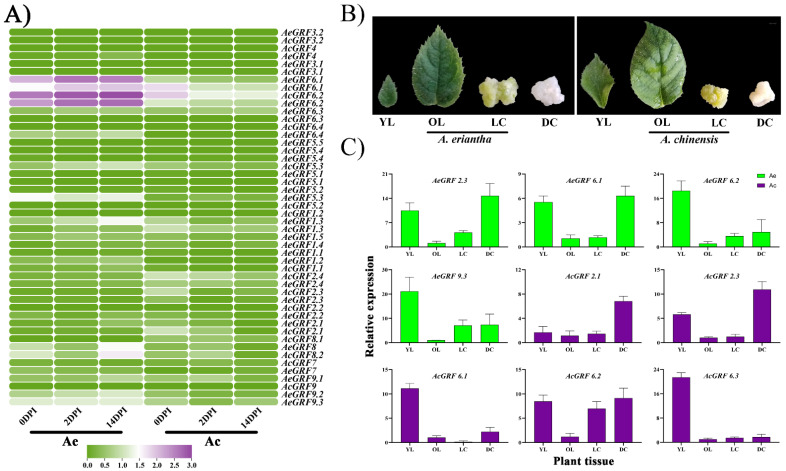
Expression analysis of *GRFs* in kiwifruit. (**A**) Heatmap for 22 *AcGRFs* and 26 *AeGRFs* under *Psa.* invasion in Ac and Ae. (**B**) Phenotype for different kiwifruit tissues used for RT-qPCR analysis. (**C**) RT-qPCR analysis for nine highly expressed genes from four different RNA-seq datasets. The YL stands for young leaves, OL stands for old leaves, LC stands for callus under light treatment, DC stands for callus under dark treatment, and DPI stands for days post-inoculation of *Psa*.

To investigate the expression analysis of kiwifruit *GRF* genes, we obtained three RNA-seq datasets from KGD and re-analyzed them. The first RNA-seq dataset was carried out in Ac and Ae under *Psa.* invasion, and in leaves, roots, and stems of Ac. Results showed 11 genes highly expressed in both kiwifruit species under *Psa.* invasion, and 12 *GRFs* significantly expressed in leaves, roots, and stems of Ac. However, the expression profile for *GRF* genes was relatively stronger in Ae compared to Ac under *Psa.* invasion (Figure 5A and Figure S5A). In the second RNA-seq dataset, the eight *GRFs* from Ac were specifically expressed in immature fruit. In contrast, *AcGRF7* and *AcGRF8.2* were highly expressed in ripen fruit of Ac. We also observed a few genes weakly expressed in mature fruit (Figure S5B). The third RNA-seq dataset consists of samples taken at different time points from Ac and Ae under *Psa.* invasion. The results showed that eight *AcGRF* transcripts were specifically expressed in samples taken from Ac and Ae (Figure S5C). Interestingly, most of the highly expressing kiwifruit *GRFs* had a close phylogenetic relationship, indicating their functional similarities in kiwifruit. We selected nine highly expressing genes commonly found in all RNA-seq datasets. Then, we did RT-qPCR for these genes to confirm their expression levels in young leaves (YL), old leaves (OL), and calluses under light (LC) and dark (DC) treatment (Figure 5B). The results showed a highly tissue-specific expression profile for selected genes. Most of the genes showed higher expression in young leaves and calluses. Interestingly, the expression profile for most of *GRFs* was relatively higher in young leaves and callus under dark conditions, indicating their vital role in plant growth and response to stress conditions (Figure 5C). 

### 2.7. Promoter Analysis in Kiwifruit GRFs

Finding cis-regulatory elements in the promoter region provide insights into the regulation of downstream genes. In the present study, we did promoter analysis in the 1000 bp upstream promoter region to find out potential cis-regulatory elements responsible for the regulation of kiwifruit *GRFs*. Our results showed seven growth and development responsive elements, seven defense responsive elements, five phytohormones responsive elements and two storage protein responsive elements in kiwifruit *GRFs*. The cis-regulatory element distribution in the upstream promoter region of nine highly expressing kiwifruit *GRFs* is given in (Table 1) and for all kiwifruit *GRFs* is given in (Figure S6). The results showed that cis-elements were randomly distributed in promoter regions of kiwifruit *GRFs*. For example, circadian cycle responsive element (CCRE) was present only in *AeGRF2.1*. Similarly, wound responsive element (WRE) was found only in *AeGRF6.2*. In contrast, light-responsive elements (LRE) were abundantly present in all kiwifruit *GRFs*.

### 2.8. Protein-Protein Association and Protein Structure Analysis

**Figure 6:**
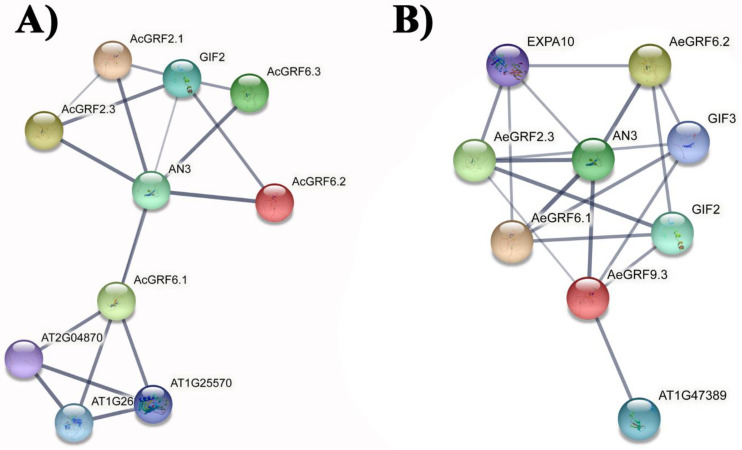
Prediction of protein-protein interaction networks for kiwifruit *GRFs* based on *A. thaliana* orthologs. (**A**) Protein-protein interaction networks for *AcGRF* proteins. (**B**) Protein-protein interaction networks for *AcGRF* proteins. Grey line thickness indicates the strength of protein-protein interaction.

STRING database was employed to predict protein-protein interaction association by finding kiwifruit *GRFs* orthologs in *A. thaliana*. Results for Ac proteins showed that *AcGRF* 2.1, 2.3, 6.1, 6.2, and 6.3 showed association with AN3 (GIF1) and GIF2 (Figure 6A). Similarly, *AeGRF* 2.3, 6.1, 6.2, and 9.3 showed association with AN3 (GIF1), GIF2, and GIF 3 (Figure 6B). These results indicated that the above-mentioned proteins have affinity to form *GRF*-GIF regulatory complex in kiwifruit. Three-dimensional structures of nine highly expressing proteins from different RNA-seq datasets were predicted and visualized by Phyre 2 online tool. The results showed highly similar structures for all selected *AcGRF* and *AeGRF* proteins, indicating their functional similarity in kiwifruit (Figure S7).

## 3. Discussion

Whole-genome sequencing has been done in many plants owing to the rapid development of sequencing technologies [28,29]. Growth-regulating factors (*GRFs*) are plant-specific regulation factors responsible for regulation of growth and development in plants under normal and stress conditions [30]. The number of *GRFs* in plants varies between 8–20, however it is less in lower plant taxa (only two *GRFs* in mosses) [31]. Due to the unavailability of reported knowledge, it was prudent to carry out identification and characterization of *GRFs* in kiwifruit.

### 3.1. Identification of Kiwifruit GRFs and Gene Structure Analysis

In the present study, we identified 22 *AcGRFs* and 26 *AeGRFs* in whole genomes of Ac and Ae, respectively. Gene structure provides valuable insights into gene functioning in plants. Previously, two highly conserved domains QLQ and WRC has been reported in N-terminal of *A. thaliana* *GRF* proteins [3]. The QLQ domain is specific for interacting with GIF, while the WRC domain acts as a transcriptional regulator by interacting with cis-regulatory elements of downstream genes. We observed some kiwifruit *GRFs* lacking one of the conserved domains, however, it will be interesting to know if *GRFs* with a single conserved domain play a role similar to *GRFs* with both conserved domains. It is believed that some less conserved domains (TQL, GGPL, and FFD) in the C-terminal possibly can play a role in functional diversification of some *GRFs* [11]; therefore, it is important to consider the C-terminal while assessing the function of kiwifruit *GRFs*. The divergence in coding or non-coding region of genes is a key step to understand their functional and evolutionary relationships [32]. Additionally, the gain or loss of introns and exons brings functional differences in genes. We observed two to four intron/exons in kiwifruit *GRFs* similar to *A. thaliana*. Almost 50% of kiwifruit *GRFs* contained three introns and four exons, indicating that kiwifruit *GRFs* have highly conserved structural evolution.

### 3.2. Phylogenetic Analysis in Kiwifruit GRFs

Previously, the different phylogenetic groups in monocots and dicots indicated the differences in evolution patterns and gene characteristics of *GRFs* in both plant groups [3,5,8,11]. We observed five distinct phylogenetic groups in kiwifruit similar to *A. thaliana* due to the dicotyledonous nature of both plants. Additionally, the phylogenetic group VI and V in kiwifruit were larger in number than other groups (I, II, and III), implying the occurrence of independent events of gene gain/loss in these groups. 

### 3.3. Gene Duplication/Deletion Analysis in Kiwifruit GRFs

The whole genomes (Ae and Ac) used in this study belong to diploid kiwifruit, and logically, they must have one extra copy of a gene for each homolog of *A. thaliana*. However, some genes in Ac only have a single copy (for example *AcGRF9*) and others have several copies in both genomes, indicating that kiwifruit genomes underwent intensive genome gain and loss events. Gene duplication events play a major role in the formation of gene families. Although the kiwifruit genomes were five times larger in size than the *A. thaliana* genome (Ae = 690.6 Mb, Ac = 616.1 Mb, and *A. thaliana* = 125 Mb) [22,24,33], the kiwifruit *GRFs* (26 members in Ae and 22 members in Ac) were only three times higher in number than *A. thaliana* (nine genes), indicating a significant amount of genome loss during duplication/deletion events in kiwifruit. Previous results indicated the occurrence of whole-genome triplication events in kiwifruit similar to the eudicot ancestors [34]. In the present study, we observed whole-genome duplication a major source of kiwifruit *GRFs* evolution. The kiwifruit *GRFs* showed strong collinearity and homology both within and between the species. Additionally, the purification selection was the main source of evolution in kiwifruit *GRFs*. The non-synonymous (Ks) mutations are a limiting factor for determining the type of selection in gene pairs. An increase or decrease in non-synonymous mutations will affect the final results. In case of our results, lower non-synonymous mutations (Ks) in ortholog and paralog gene pairs played a key role in identifying the purification selection that occurred in them. The above-mentioned phenomena indicated that kiwifruit *GRFs* followed a highly conservative evolution model.

### 3.4. Gene Expression Analysis

Previously, researchers reported higher expression levels of plant *GRFs* in actively developing tissues than in mature tissues [31,35]. For example, altered expression of *AtGRFs* caused significant changes in growth phenotypes of plants [11,36,37]. Similarly, the application of plant growth hormones and various stress conditions also altered the expression profile of *GRFs* in plants [1,38,39]. In the present study, higher expression levels of kiwifruit *GRFs* in immature tissue and under *Psa.* invasion indicated their regulatory role in plant growth, development, and stress tolerance. The presence of stress and growth-related cis-regulatory element in the promoter region of kiwifruit *GRFs* complimented their higher expression levels in transcriptomic data. Similarly, the higher expression levels of selected kiwifruit *GRFs* observed by RT-qPCR in actively dividing cells of callus and young leaves not only validated RNA-seq data but supported our argument about their role in the growth and development of kiwifruit. Additionally, higher expression levels of genes in callus under dark conditions confirm their role in shade stress similar to the previous findings in soybean [9].

To summarize, we identified *GRF* family members in whole genomes of Ac and Ae. A series of analyses was carried out to characterize the *GRF* family members in both kiwifruit genomes. We found higher expression levels for two genes from Ac (*AcGRF6.1* and *AcGRF6.2*) and two genes from Ae (*AeGRF6.1* and *AeGRF6.2*) in the different transcriptomic datasets, which can be a valuable source for future studies. Further investigations are required to functionally characterize these genes in kiwifruit for growth, development, and stress responses. 

## 4. Materials and Methods

### 4.1. Gene Identification

The protein sequences and genome annotations for *AcGRF* and *AeGRF* were retrieved from the Kiwifruit Genome Database (KGD) [40]. The *AtGRF* protein sequences were retrieved from The *Arabidopsis* Information Resources (TAIR) [41] and used to predict *GRF* proteins in KGD, based on their homology. The resulting protein sequences were used to construct the kiwifruit-specific HMM model for identification of *AcGRFs* and *AeGRFs* proteins by HMMER 3.0 software [42]. The conserved domains were verified by the Conserved Domain Database (CDD) [43], and the Simple Modular Architecture Research Tool (SMART) [44]. The predicted proteins were considered as *AcGRFs* and *AeGRFs* only if they contained one or both QLQ (PF0889) and WRC (PF0880) conserved domains.

### 4.2. Gene Structure Analysis for Kiwifruit GRFs

The genomic and coding sequences for kiwifruit *GRFs* were retrieved from KGD [40]. The protein length, molecular weight (MW), isoelectric point (theoretical pI), and grand average of hydropathicity (GRAVY) were computed by ExPASy server [45]. The exon-intron distribution patterns of kiwifruit *GRFs* were investigated by Gene Structure Display Server (GSD 2.0) [46]. The conserved motifs were predicted by MEME software [47] with a maximum number of 15 motifs. The chromosome length and the chromosomal distribution of genes were retrieved from KGD. 

### 4.3. Multiple Sequence Alignments and Phylogenetic Analysis

The multiple sequence alignment of *AtGRFs*, *AcGRFs*, and *AeGRFs* proteins was performed by ClustalX with default parameters [48]. The phylogenetic tress was built with GenomeNet Database Resources (https://www.genome.jp/, accessed on 10 December 2021) by using a phylogenetic analysis pipeline from ETE3 with default parameters (Aligner = mafft_default, Alignment cleaner = none, Model tester = none, and Tree builder = iqtree_default), and visualized by iTOOLs software [49]. The sub-cellular localization of the candidate protein was predicted by CELLO software [50].

### 4.4. Gene Duplication and Evolution Analysis

The kiwifruit genome of Ac and Ae were subjected to an all-to-all blast by Blastp, and the MCScan program with default parameters was used to analyze the duplication events in kiwifruit *GRFs* [51]. The duplicated *GRF* gene pairs were subjected to TBtools software for calculation of synonymous (Ka) and non-synonymous (Ks) substitution rates [52]. The divergence time was calculated by following formula: T = (Ks/2r) × 10^−6^, where Ks is the non-synonymous substitution rate, T stands for divergence time, and r denotes the neutral substitution rate (r = 3.39 × 10^−9^). The resulting values were divided by 1 million (10^−6^) to convert them into million years [25]. The collinearity blocks across the whole genome were produced by using MCScan software with default parameters [51]. The paralog and ortholog gene pairs were visualized by using TBtools software [52]. 

### 4.5. Cis-Regulatory Elements Analysis in the Upstream Promoter Region and of Kiwifruit GRFs

We retrieved 1000 bp upstream sequences of kiwifruit *GRFs* as the promoter region from KGD [40]. The cis-regulatory elements were predicted by the PlantCARE database [53] and visualized by TBtools software [52]. 

### 4.6. Protein-Protein Association Networks and Protein Structure Analysis for Kiwifruit GRFs 

The protein association network analysis for kiwifruit *GRFs* was done by using STRING database [54]. Similarly, the protein structures for kiwifruit *GRFs* were predicted by Phyre 2 online tool [55] and visualized by JSmol interactive viewer [56].

### 4.7. Expression Analysis of Kiwifruit GRFs

We downloaded three published RNA-seq datasets (PRJNA187369, PRJNA328414, and PRJNA514180) from NCBI (National Center for Biotechnology Information) to investigate the expression profile of kiwifruit *GRFs*. The datasets were re-analyzed against reference genomes of “White” and “Hong Yang” cultivars [24,25]. The HISAT2 (v2.0.1) software was used to perform the alignment for reads [57]. The transcript assembly and quantification were performed by STRINGTIE (v2.1.5) [58]. The FPKM values for kiwifruit *GRFs* were used to draw heatmaps.

### 4.8. RT-qPCR Analysis

Plant samples for RT-qPCR were taken from young leaves (YL), old leaves (OL), callus under light conditions (LC), and callus under dark condition (DC) of Ac and Ae. A HiPure plant RNA mini kit was used to extract total RNA by following manufacturer protocol (Angen Biotech, Guangzhou, China). The RNA degradation and contamination were determined by subjecting samples to agarose gel electrophoresis, and RNA purity was tested on a NanPhotometer^®^ (Huake, Zhejiang, China) by using OD_260_/OD_280_. Similarly, the cDNA was synthesized by TransScript^®^ One-Step gDNA Removal and cDNA Synthesis SuperMix kit by following the manufacturer protocol (TransGen Biotech, Beijing, China). The reaction mixture was prepared by using a *PerfectStart*^TM^ Green qPCR SuperMix kit by following the given instructions ((TransGen Biotech, Beijing, China)). The qRT-PCR was performed on a CFX Connect Real-Time PCR Detection System (BIO-RAD, Hercules, CA, USA). The reactions were prepared in a total volume of 20 µL containing: 1 µL of template, 10 µL of MonAMP^TM^ ChemoHS qPCR Mix, 0.5 µL of each specific primer. The following conditions were set to perform the reactions: initial denaturation step at 95 °C for 5 min followed by 45 cycles of 95 °C for 10 sec, 60 °C for 20 sec, and 72 °C for 20 sec [59]. Thekiwifruit *β-actin* gene was used as an internal control for normalization [60]. All the reactions were replicated thrice. The relative expression was calculated by 2^−ΔΔCt^ method [61]. Primer pairs used in this study are listed in Table S5.

## Figures and Tables

**Table 1:** Prediction of cis-regulatory elements in the upstream promoter region of nine highly expressing kiwifruit *GRFs* from different RNA-seq datasets of kiwifruits. A list of full names for acronyms is added in Table S4.

Cis-Element		Gene Name								
Group	Name	*AcGRF*					*AeGRF*			
		*2.1*	*2.3*	*6.1*	*6.2*	*6.3*	*2.1*	*6.1*	*6.2*	*9.3*
**Growth and development**	CCRRE									
	CCRE						1			
	SSRRE									
	MERE	1				1		1		
	EERE									
	DPMCRE									
	LRE	3	4	10	3	5	6	3	3	6
**Plant defense**	DSRE									
	LTRE									
	AIRE	1	2			2	2			1
	MEMARE		1							
	ASIRE	1	1							
	DIRE				1				1	1
	WRE								1	
	FBGRRE									
**Phytohormones**	GARE				1		2			
	SARE	1								
	AuxRE		1		1		1			
	MeJARE	4		4				4		
	ABARE			3	1			1		3

## Data Availability

The data supporting reported results can be found at https://kiwifruitgenome.org/.

## Supplementary Materials

The following supporting information can be downloaded at: https://www.mdpi.com/article/10.3390/plants11131633/s1, Figure S1. Multiple sequence alignment of *AcGRF* and *AeGRF* proteins. Identical amino acids (aa) are indicated by colored background. The QLQ and WRC domains are presented by colored rectangle boxes. Figure S2. Number of genes on each chromosome of *Ac* and *Ae*. Figure S3. Occurrence of exons and introns in *AcGRF* and *AeGRF* genes. Figure S4. Logos for conserved motifs in *AcGRF* and *AeGRF* proteins. Figure S5. Heatmaps for different transcriptomic data of kiwifruit. (A) Heatmap showing expression profile of *AcGRFs* and *AeGRFs* in different plant tissues of *Ac* under *Psa.* invasion. (B) Heatmap representing expression profile of *AcGRFs* and *AeGRFs* in *Ac* and *Ae* under *Psa.* invasion. (C) Expression profile of *AcGRFs* in fruit samples of *Ac* taken at different developmental stages. Figure S6. Promoter analysis of *AcGRFs* and *AeGRFs* for cis-regulatory elements. Figure S7. Protein structure analysis for *AeGRFs* and *AcGRFs*. Table S1. Characterization of kiwifruit *GRFs*. Table S2. Collinearity analysis of kiwifruit *GRFs*. Table S3. Sequences for motifs predicted in kiwifruit *GRFs*. Table S4. Detail for acronyms used in cis-regulatory elements in Table 1. Table S5. List of pair of primers used in RT-qPCR analysis.
