# Supplementary material for: Genome-Wide Identification and Structural Characterization of Growth-Regulating Factors (GRFs) in Actinida eriantha and Actinidia chinensis
Source: Plants (Basel). 2022 Jun 21;11(13):1633. doi: 10.3390/plants11131633 (PMC9269249; doi:10.3390/plants11131633)
Supplement: Supplementary file 1 [file plants-11-01633-s001.zip › plants-1664404-supplementary.pdf]

Supplementary Files

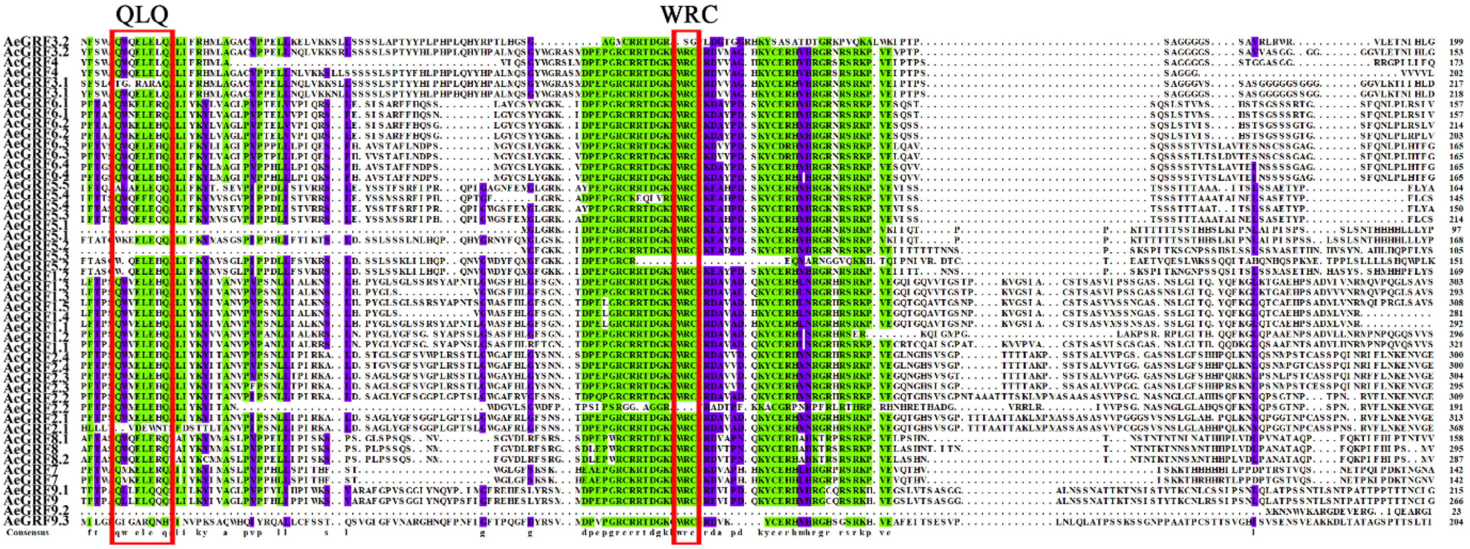

Figure S1. Multiple sequence alignment of *Ac*GRF and *Ae*GRF proteins. Identical amino acids (aa) are indicated by colored background. The QLQ and WRC domains are presented by colored rectangle boxes.

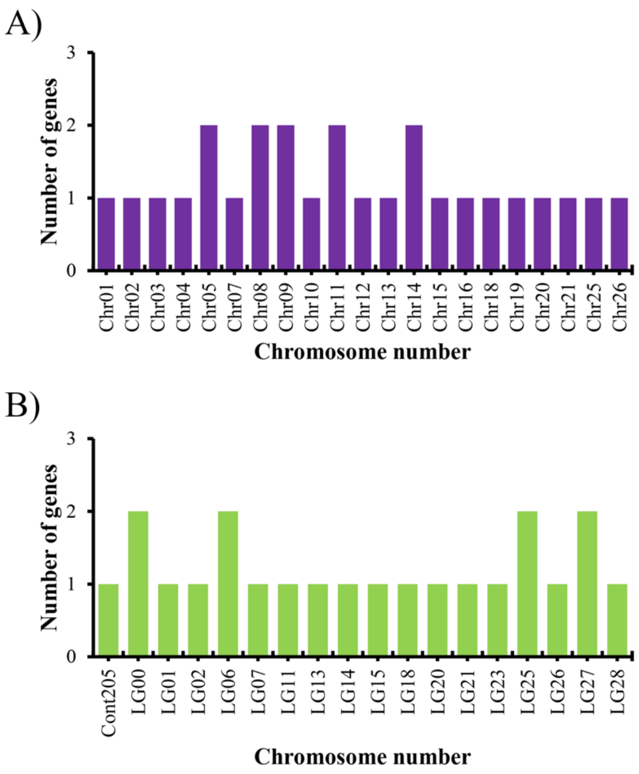

Figure S2. Number of kiwifruit *GRF* genes on each chromosome of A) *Ac* and B) *Ae*.

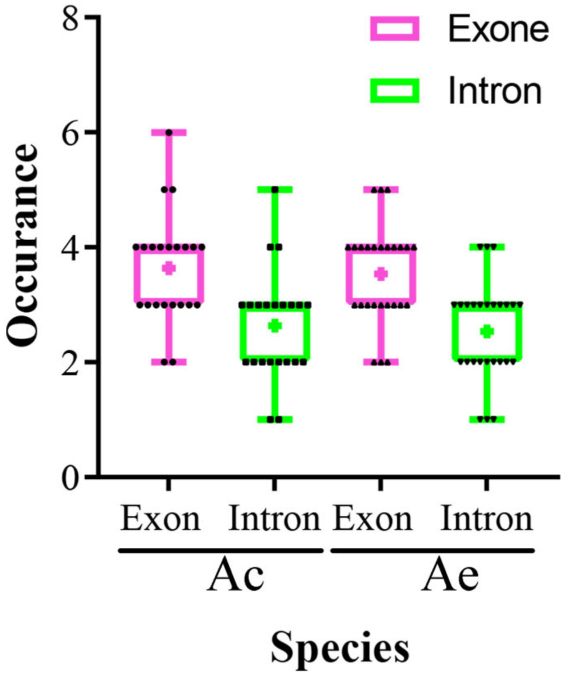

Figure S3. Occurrence of exons and introns in *AcGRFs* and *AeGRFs* genes.

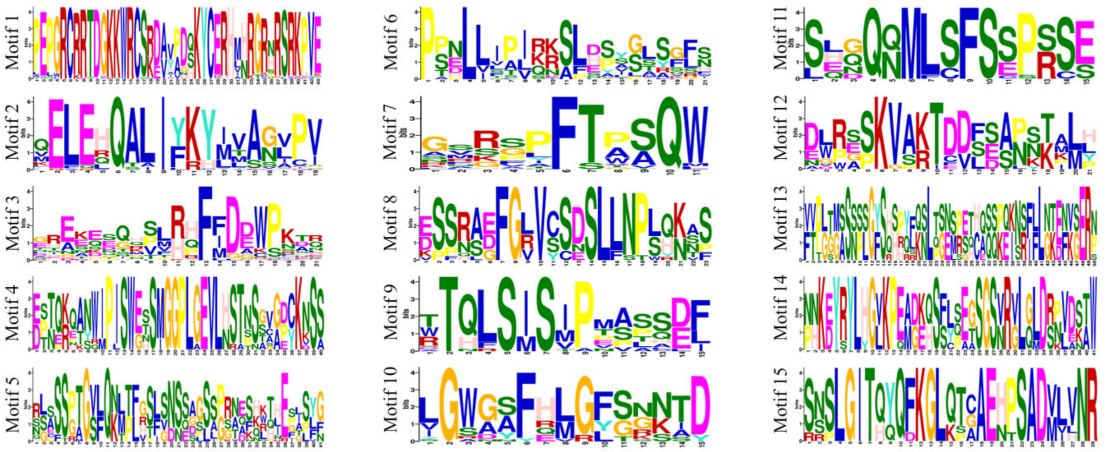

Figure S4. Logos for conserved motifs in *Ac*GRFs and *Ae*GRFs proteins.

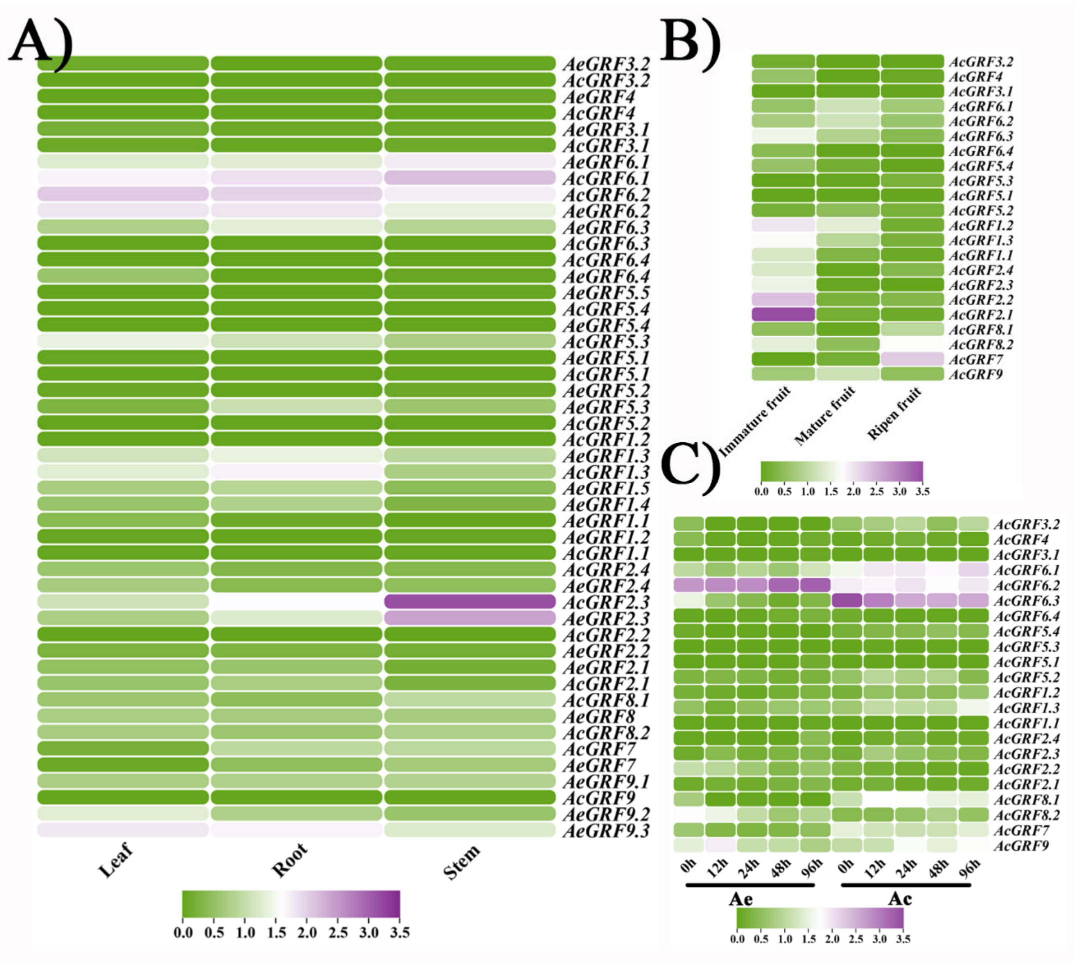

Figure S5. Heatmaps for different transcriptomic data of kiwifruit. **A)** Heatmap showing expression profile of *AcGRFs* and *AeGRFs* in different plant tissues of *Ac* under *Psa.* invasion**.** **B)** Expression profile of *AcGRFs* in fruit samples of *Ac* taken at different developmental stages. **C)** Heatmap representing expression profile of *AcGRFs* and *AeGRFs* in *Ac* and *Ae* under *Psa.* invasion.

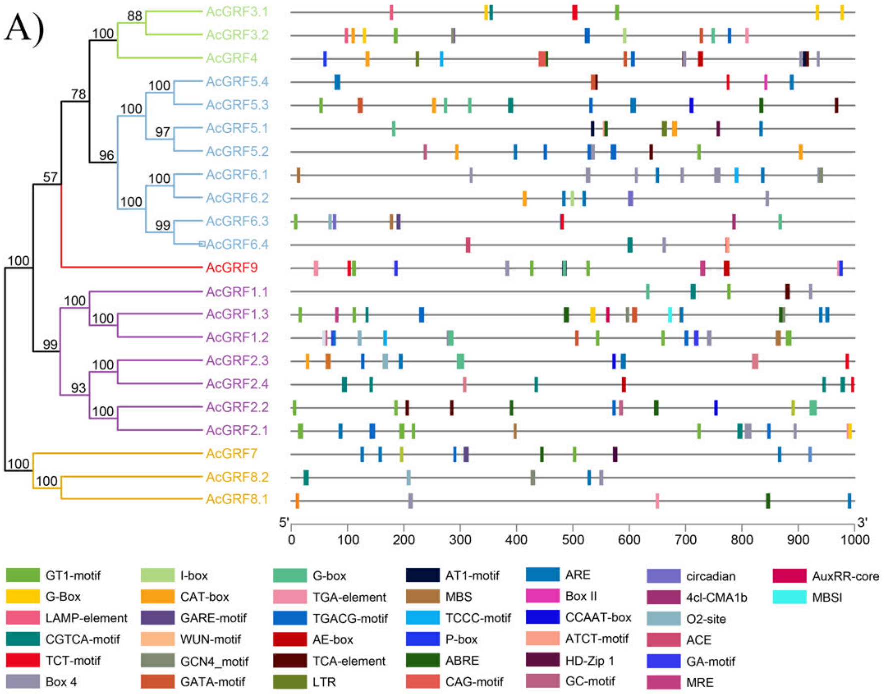

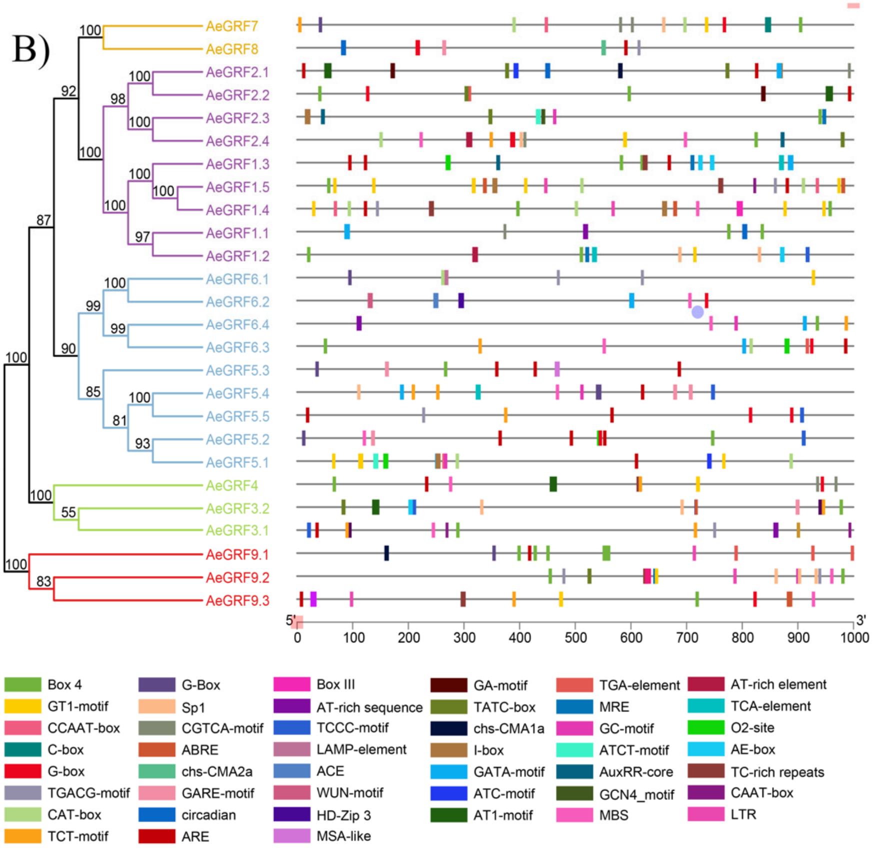

Figure S6. Promoter analysis of *AcGRFs* and *AeGRFs* for cis-regulatory elements.

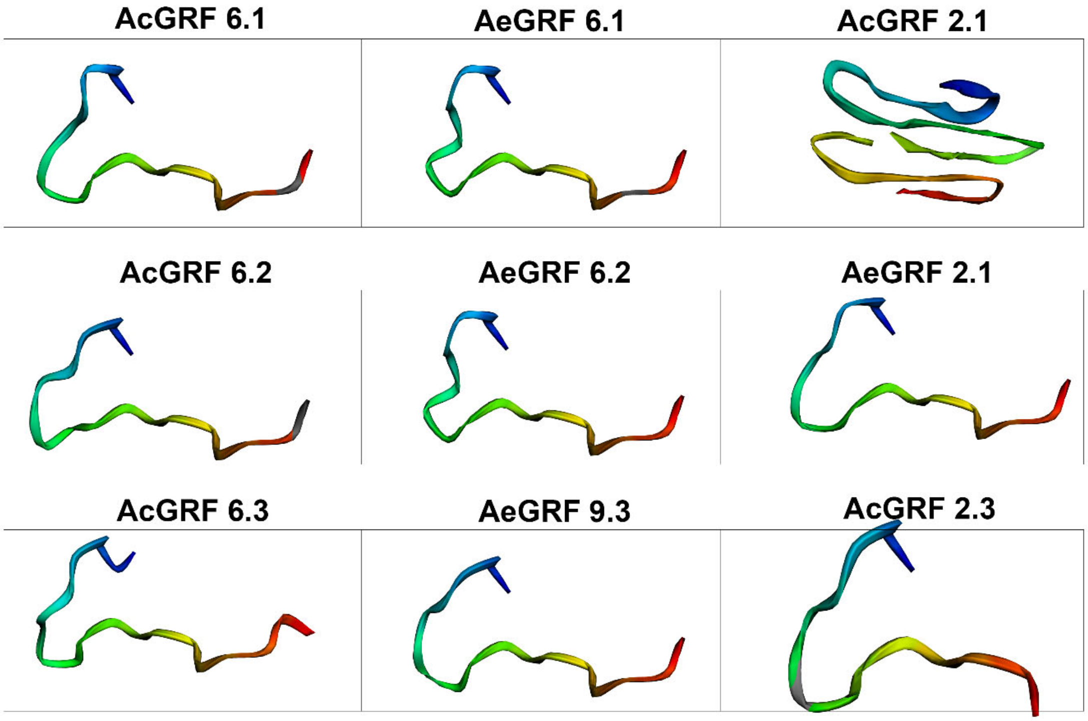

Figure S7. Protein structure analysis for *Ae*GRFs and *Ac*GRFs.

Table S1. Characterization of kiwifruit GRFs

Gene ID	Name	CDS (bp)	Genomic sequence (bp)	Protein (aa)	MW (kDa)	pI	GRAVY	II	Protein stability
**Actinidia16410**	*AcGRF1.1*	1719	4538	572	62.20	7.97	-0.603	57.67	Unstable
**Actinidia34541**	*AcGRF1.2*	1683	5246	560	60.53	8.39	-0.569	41.72	Unstable
**Actinidia11963**	*AcGRF1.3*	1584	5339	527	57.36	8.95	-0.563	38.14	Stable
**Actinidia19587**	*AcGRF2.1*	1773	13127	590	61.41	6.56	-0.443	52.14	Unstable
**Actinidia29634**	*AcGRF2.2*	1632	4097	543	58.28	9.04	-0.541	44.24	Unstable
**Actinidia10222**	*AcGRF2.3*	1596	3458	531	57.93	8.72	-0.6	47.2	Unstable
**Actinidia19295**	*AcGRF2.4*	1167	3204	388	43.04	8.75	-0.708	46.11	Unstable
**Actinidia10395**	*AcGRF3.1*	1179	3665	392	42.34	6.76	-0.743	55.44	Unstable
**Actinidia34353**	*AcGRF3.2*	990	3292	329	35.12	8.84	-0.615	59.31	Unstable
**Actinidia31283**	*AcGRF4*	1011	4038	336	36.66	9.4	-0.65	56.17	Unstable
**Actinidia29924**	*AcGRF5.1*	933	2053	310	35.45	8.98	-0.938	55.03	Unstable
**Actinidia30207**	*AcGRF5.2*	993	3330	330	37.73	8.38	-0.889	56.17	Unstable
**Actinidia21789**	*AcGRF5.3*	1059	1625	352	39.82	9.44	-0.794	68.57	Unstable
**Actinidia32187**	*AcGRF5.4*	867	2226	288	32.52	8.68	-0.827	65.47	Unstable
**Actinidia18155**	*AcGRF6.1*	993	7499	330	36.87	7.59	0.77	60.38	Unstable
**Actinidia14749**	*AcGRF6.2*	1161	9184	386	43.26	9.11	-0.831	63.27	Unstable
**Actinidia33616**	*AcGRF6.3*	894	8975	297	33.24	6.83	-0.708	47.24	Unstable
**Actinidia33799**	*AcGRF6.4*	588	1992	195	21.55	9.21	-0.606	49.43	Unstable
**Actinidia08689**	*AcGRF7*	996	1615	331	36.95	5.92	-0.575	49.55	Unstable
**Actinidia04172**	*AcGRF8.1*	1029	3106	342	38.40	7.67	-0.819	54.96	Unstable
**Actinidia22212**	*AcGRF8.2*	1620	3717	539	58.32	6.31	-0.747	52.58	Unstable
**Actinidia31428**	*AcGRF9*	1122	3344	373	39.80	9.7	-0.276	42.34	Unstable
**DTZ79_01g06110**	*AeGRF1.1*	1644	4884	547	59.45	6.63	-0.593	56.93	Unstable
**DTZ79_09g04950**	*AeGRF1.2*	690	10476	229	24.78	9.19	-0.669	53.37	Unstable
**DTZ79_13g09430**	*AeGRF1.3*	1263	5258	420	45.88	8.92	-0.659	41.45	Unstable
**DTZ79_05g09790**	*AeGRF1.4*	879	5152	292	31.73	9.4	-0.615	43.15	Unstable
**DTZ79_05g12280**	*AeGRF1.5*	846	5132	281	30.56	9.32	-0.605	39.15	Stable
**DTZ79_11g02690**	*AeGRF2.1*	1608	5203	535	57.54	8.88	-0.554	47.03	Unstable
**DTZ79_10g09990**	*AeGRF2.2*	1275	3875	424	45.65	9.22	-0.585	52.59	Unstable
**DTZ79_21g04810**	*AeGRF2.3*	1569	3752	522	57.05	8.81	-0.599	50.65	Unstable
**DTZ79_04g04500**	*AeGRF2.4*	1578	3671	525	57.38	8.39	-0.623	47.27	Unstable
**DTZ79_08g01320**	*AeGRF3.1*	1149	3859	382	41.30	9.4	-0.655	57.87	Unstable
**DTZ79_19g02440**	*AeGRF3.2*	831	3828	276	30.36	9.49	-0.523	62.43	Unstable
**DTZ79_15g05950**	*AeGRF4*	687	4212	228	25.59	9.34	-0.366	65.71	Unstable
**DTZ79_09g00650**	*AeGRF5.1*	768	2106	256	29.19	9.23	-1.064	49.44	Unstable
**DTZ79_12g00680**	*AeGRF5.2*	522	2375	173	19.42	9.01	-1.179	50.69	Unstable
**DTZ79_20g15170**	*AeGRF5.3*	939	2391	312	35.68	7.7	-0.425	47.25	Unstable
**DTZ79_16g01240**	*AeGRF5.4*	852	1124	283	32.68	9.55	-0.766	68.25	Unstable
**DTZ79_11g10240**	*AeGRF5.5*	912	1817	303	33.87	9.08	-0.866	58.27	Unstable
**DTZ79_26g03100**	*AeGRF6.1*	993	5807	330	36.90	8.39	-0.757	61.84	Unstable
**DTZ79_25g08890**	*AeGRF6.2*	813	9674	270	30.39	9.69	-0.712	62.01	Unstable
**DTZ79_03g00230**	*AeGRF6.3*	948	9677	315	35.02	6.8	-0.671	48.38	Unstable
**DTZ79_02g00190**	*AeGRF6.4*	1065	6766	354	38.88	8.45	-0.6	50.5	Unstable

Table S1. Characterization of kiwifruit GRF (cont.)

Gene ID	Name	CDS (bp)	Genomic sequence (bp)	Protein (aa)	MW (kDa)	pI	GRAVY	II	Protein stability
DTZ79_18g07490	*AeGRF7*	996	1191	331	37.20	5.8	-0.621	49.96	Unstable
DTZ79_14g02820	*AeGRF8*	1644	3923	547	59.05	6.23	-0.746	52.38	Unstable
DTZ79_08g09020	*AeGRF9.1*	1497	4518	498	53.61	9.6	-0.359	50.13	Unstable
DTZ79_14g09470	*AeGRF9.2*	1305	4093	434	46.94	9.41	-0.619	54.06	Unstable
DTZ79_07g05060	*AeGRF9.3*	624	2508	207	23.32	8.92	-0.312	40.28	Unstable

Note: CDS= Coding sequence, MW= molecular weight, pI= theoretical isometric point, GRAVY= grand average of hydropathicity, and II = instability index.

Table S2. Collinearity analysis of kiwifruit GRFs.

Sequence 1	Sequence 2	Ka	Ks	Ka/Ks	MYA
**Paralog gene pairs in *A. chinensis***					
AcGRF3.1	***AcGRF4***	0.11	0.33	0.34	49.41
AcGRF5.3	***AcGRF5.4***	0.06	0.10	0.59	15.25
AcGRF8.1	***AcGRF8.2***	0.06	0.15	0.44	21.73
AcGRF2.2	***AcGRF2.1***	0.10	0.34	0.29	50.87
AcGRF6.1	***AcGRF6.2***	0.03	0.15	0.22	22.03
AcGRF2.3	***AcGRF2.4***	0.04	0.15	0.28	21.95
AcGRF3.1	***AcGRF4***	0.11	0.33	0.34	59.90
**Paralog gene pairs in *A. eriantha***					
AeGRF6.4	***AeGRF6.3***	0.05	0.14	0.40	19.97
AeGRF2.4	***AeGRF2.3***	0.05	0.18	0.31	26.04
AeGRF5.1	***AeGRF5.2***	0.17	0.54	0.31	79.55
AeGRF1.4	***AeGRF1.3***	0.06	0.17	0.36	24.94
AeGRF6.2	***AeGRF6.1***	0.04	0.16	0.27	23.15
**Ortholog gene pairs between *A. erintha* and *A. chinensis***					
AeGRF1.1	***AcGRF1.1***	0.085091	0.172223	0.494075	25.40
AeGRF6.4	***AcGRF6.4***	0.004474	0.022279	0.200831	3.29
AeGRF6.3	***AcGRF6.4***	0.0366	0.135557	0.269995	19.99
AeGRF2.4	***AcGRF2.3***	0.039061	0.154338	0.253084	22.76
AeGRF2.4	***AcGRF2.4***	0.015886	0.041364	0.384046	6.10
AeGRF1.4	***AcGRF1.3***	0.013854	0.027738	0.499444	4.09
AeGRF3.1	***AcGRF3.1***	0.115135	0.191248	0.602022	28.21
AeGRF9.1	***AcGRF9***	0.041328	0.097711	0.422963	14.41
AeGRF5.1	***AcGRF5.1***	0.039403	0.042486	0.927436	6.27
AeGRF1.2	***AcGRF1.1***	0.058306	0.088007	0.662514	12.98
AeGRF2.2	***AcGRF2.2***	0.093082	0.209972	0.443307	30.97
AeGRF5.5	***AcGRF5.3***	0.106539	0.157851	0.674936	23.28
AeGRF2.1	***AcGRF2.2***	0.055359	0.229352	0.241371	33.83
AeGRF2.1	***AcGRF2.1***	0.059867	0.14249	0.420145	21.02
AeGRF5.5	***AcGRF5.4***	0.039874	0.038802	1.027631	5.72
AeGRF1.3	***AcGRF1.3***	0.074736	0.206702	0.361562	30.49
AeGRF1.3	***AcGRF1.2***	0.028721	0.073208	0.392316	10.80
AeGRF8	***AcGRF8.2***	0.004811	0.033816	0.142275	4.99
AeGRF5.4	***AcGRF5.3***	0.070526	0.107069	0.658698	15.79
AeGRF5.4	***AcGRF5.4***	0.12392	0.169619	0.73058	25.02
AeGRF7	***AcGRF7***	0.025129	0.055538	0.452466	8.19
AeGRF2.3	***AcGRF2.3***	0.059435	0.090992	0.653188	13.42
AeGRF2.3	***AcGRF2.4***	0.05955	0.167605	0.355301	24.72
AeGRF6.2	***AcGRF6.1***	0.046835	0.171782	0.272642	25.34
AeGRF6.2	***AcGRF6.2***	0.030163	0.076735	0.393086	11.32
AeGRF6.1	***AcGRF6.1***	0.014586	0.049754	0.293161	7.34
AeGRF6.1	***AcGRF6.2***	0.029624	0.127918	0.231581	18.87

Table S3. Sequences for motifs predicted in kiwifruit GRFs.

Motif number	Sequence
1	PEPGRCRRTDGKKWRCSRDAVPDQKYCERHMHRGRNRSRKPVE
2	QELEHQALIYKYIVAGVPV
3	GREKZSQHSLRHFFDDWPKTQ
4	EPTQKQANWIPISWESSMGGPLGEVLHSTNSGVGDCKNSS
5	RLASSPTGVLQKLTFGSLSNSSAGSSPRNESHKTHESLSYG
6	PPNLLIPIRKSLDSSGLSGFS
7	GSRSPFTPSQW
8	ESSRAEFGLVCSDSLLNPLQKAS
9	TTQLSISIPMASSDF
10	LGWGSFHLGFSNNTD
11	SEGQNMLSFSSPRSE
12	DLRSSKVAKTDDFSAPSTALH
13	VVPGTGASNPLGYSHHPYFKNLTGESPETHQSSPZKNRFLLGTEFKGERN
14	NNKEYRYLHGVKPEADKQSFLSEGSGSVRVLGLDRPVDSTW
15	SNSLGITQYQFKGLQTCAEHPSADVLVNR

Table S4. Detail for acronyms used in cis-regulatory elements Table 1.

Sr. No.	Acronym	Full name
1	CCRRE	Cell cycle regulation responsive element
2	CCRE	Circadian control responsive element
3	SSRRE	Seed-specific regulation responsive element
4	MERE	Meristem expression responsive element
5	EERE	Endosperm expression responsive element
6	PMCDRE	Palisade mesophyll cells responsive element
7	LRE	Light responsive element
8	DSRE	Defense and stress responsiveness responsive element
9	LTRE	Low-temperature responsiveness responsive element
10	AIRE	Anaerobic induction responsive element
11	MEMARE	Maximal elicitor-mediated activation responsive element
12	ASIRE	Anoxic specific inducibility responsive element
13	DIRE	Drought-inducibility responsive element
14	WRE	Wound responsive element
15	FBGRRE	Flavonoid biosynthetic genes regulation responsive element
16	GARE	Gibberellin-responsive element
17	SARE	Salicylic acid responsive element
18	AuxRE	Auxin responsive element
19	MeJARE	MeJA-responsive element
20	ABARE	Abscisic acid responsive element

Table S5. List of primer pairs used in RT-qPCR analysis.

Sr. No	Gene name	Forward primer	Reverse primer
**1**	***Actin***	GCTTACAGAGGCACCACTCAACC	CCGGAATCCAGCACCAATACCAG
**2**	***AcGRF2.1***	AGGTGGTGGTGGTGGGAGGTT	CACGTTCACTCTTTTTGAAAAT
**3**	***AcGRF2.3***	GTAGAAATACTGGTACGTGACAAGT	GTACTGAAGAATCAAAACCCAAACG
**4**	***AcGRF6.1***	CACCAATCGAACTGTAAGAAAGCAA	GAGAGAGAGAGAGAGAGTACAACCT
**5**	***AcGRF6.2***	CGGGCTGTAAGAATGCAAAACC	CGGCAGTGATAGAGTATCGAAGAAC
**6**	***AcGRF6.3***	CCCTCCAGTAACACGATCCTCT	GAAAATCACCACCCGCTGCTGC
**7**	***AeGRF2.3***	GTAGAAATACTGGTACGCGACAAGT	CACGAAAAGACCCTTAAGTAGTACT
**8**	***AeGRF6.1***	CGAGCTGTAAGAAAGCAAAACCTC	CAGAGAGAGAGTACAACCTAAATCG
**9**	***AeGRF6.2***	GCTTCTTGAGCTTGAGGTGATTTTC	GTGCTACCAAAGACCAAGAGTACTA
**10**	***AeGRF9.3***	CACTTGTCGCTTGGTCTATCTCTTC	TTCAAAAACCCACAGATTTCCCAGC
